# The S1Q3T3 Electrocardiographic Abnormality as a Result of Massive Empyema due to Pyogenic Liver Abscess: A Case Report

**DOI:** 10.1002/ccr3.70191

**Published:** 2025-02-07

**Authors:** Mahmonir Mohammadi, Mahnaz Valizadeh, Nasrin Rahmani‐ju, Yasamin Khosravaninezhad

**Affiliations:** ^1^ Department of Cardiology, Faculty of Medicine, Tehran Medical Sciences Islamic Azad University Tehran Iran; ^2^ Department of Internal Medicine, Faculty of Medicine, Tehran Medical Sciences Islamic Azad University Tehran Iran; ^3^ Department of Surgery, Faculty of Medicine, Tehran Medical Sciences Islamic Azad University Tehran Iran; ^4^ General Practitioner, Tehran Medical Sciences Islamic Azad University Tehran Iran

**Keywords:** ECG abnormality, liver abscess, pyogenic pleural effusion, S1Q3T3

## Abstract

The S1Q3T3 pattern on the electrocardiogram is often associated with right heart strain in pulmonary embolism, pneumothorax, and acute lung diseases causing acute cor pulmonale. The S1Q3T3 pattern during acute massive empyema in the setting of a liver abscess has not been reported in theliterature. We present a case report of a 41‐year‐old Iranian female patient with a sudden onset chest pain, dyspnea, sinus tachycardia, and electrocardiography findings of S1Q3T3 pattern, who was revealed to have an expanding pyogenic pleural effusion as a result of trans‐diaphragmatic extension of a liver abscess. The other causes of S1Q3T3 were excluded. The patient underwent the insertion of a chest tube to remove the pus. After chest tube insertion, the S1Q3T3 pattern resolved. Despite initial improvement, the patient developed complications, including pleural adhesions and lung collapse, and required a posterolateral thoracotomy with decortication. This resulted in dramatic clinical improvement. Rapidly expanding massive pyogenic pleural effusion can lead to the S1Q3T3 pattern on the electrocardiogram.

## Graphical abstract

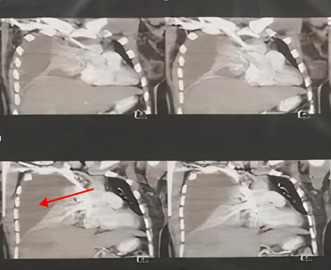

**Summary**

- Massive pyogenic pleural effusion secondary to a liver abscess can mimic the S1Q3T3 pattern, commonly linked to pulmonary embolism.
- This rare presentation highlights the need for careful differential diagnosis in patients with acute chest pain and respiratory distress to prevent misdiagnosis and ensure timely, appropriate treatment.

**Abbreviations**
CXR: chest X‐ray
ICU: intensive care unit
PLA: pyogenic liver abscess
RBBB: right bundle branch block
RUQ: right upper quadrant
VBG: venous blood gas

## 1 Introduction

S1Q3T3 pattern is a classical electrocardiographic feature of pulmonary embolism that was first described in 1935. The S1Q3T3 pattern is described as an S wave in lead I, a Q wave, and an inverted T wave in lead III [1]. The pattern is often associated with increased volume and pressure within the right ventricle due to pulmonary hypertension [2]. Pulmonary embolism is the most common diagnosis that comes to mind when realizing this pattern [3]. It may be observed in some other conditions causing pressure on the right ventricle, such as acute bronchospasm, acute lung injury, pneumothorax, ARDS, ischemic heart disease, acute and chronic cor pulmonale, cardiac tamponade, and aortic dissection [4, 5, 6]. Influenza has been associated with an increased risk of developing pyogenic empyema. The relationship between influenza and the S1Q3T3 pattern is less well described [7].

Empyema is characterized by pus accumulation in the pleural cavity. Up to 40% of empyema may be secondary to a non‐pneumonic process, such as systemic infection with hematogenous spread and/or direct inoculation [8]. Empyema is a relatively rare condition [9]. The symptoms of empyema can vary depending on the underlying cause and the individual patient. The common symptoms are chest pain, fever, dyspnea, cough, excessive sweating, fatigue, and abdominal pain [10]. Some potential complications of empyema include pleural thickening, lung abscess, sepsis, mediastinal involvement, and death. Early recognition and appropriate management of empyema are essential to prevent these complications and improve patient outcomes. Empyema can be associated with high morbidity and mortality rates, especially if left untreated or if there are underlying comorbidities [11]. It is usually diagnosed by imaging and analysis of the pleural fluid. No studies mention the accompanying empyema with the S1Q3T3 pattern imitating pulmonary embolism.

In summary, the relationship between the S1Q3T3 pattern and empyema is not well established. This study reports a patient with sudden onset chest pain, dyspnea, sinus tachycardia, and an ECG finding of the S1Q3T3 pattern due to an expanding pyogenic pleural effusion as a result of transdiaphragmatic extension of a liver abscess. Further research is needed to elucidate the relationship between the S1Q3T3 pattern and massive empyema.

## 2 Case History

A 41‐year‐old Iranian female patient presented with sudden onset chest pain, progressive shortness of breath, chest tightness, cold sweating, nausea, and vomiting on April 27, 2020.

**FIGURE 1:**
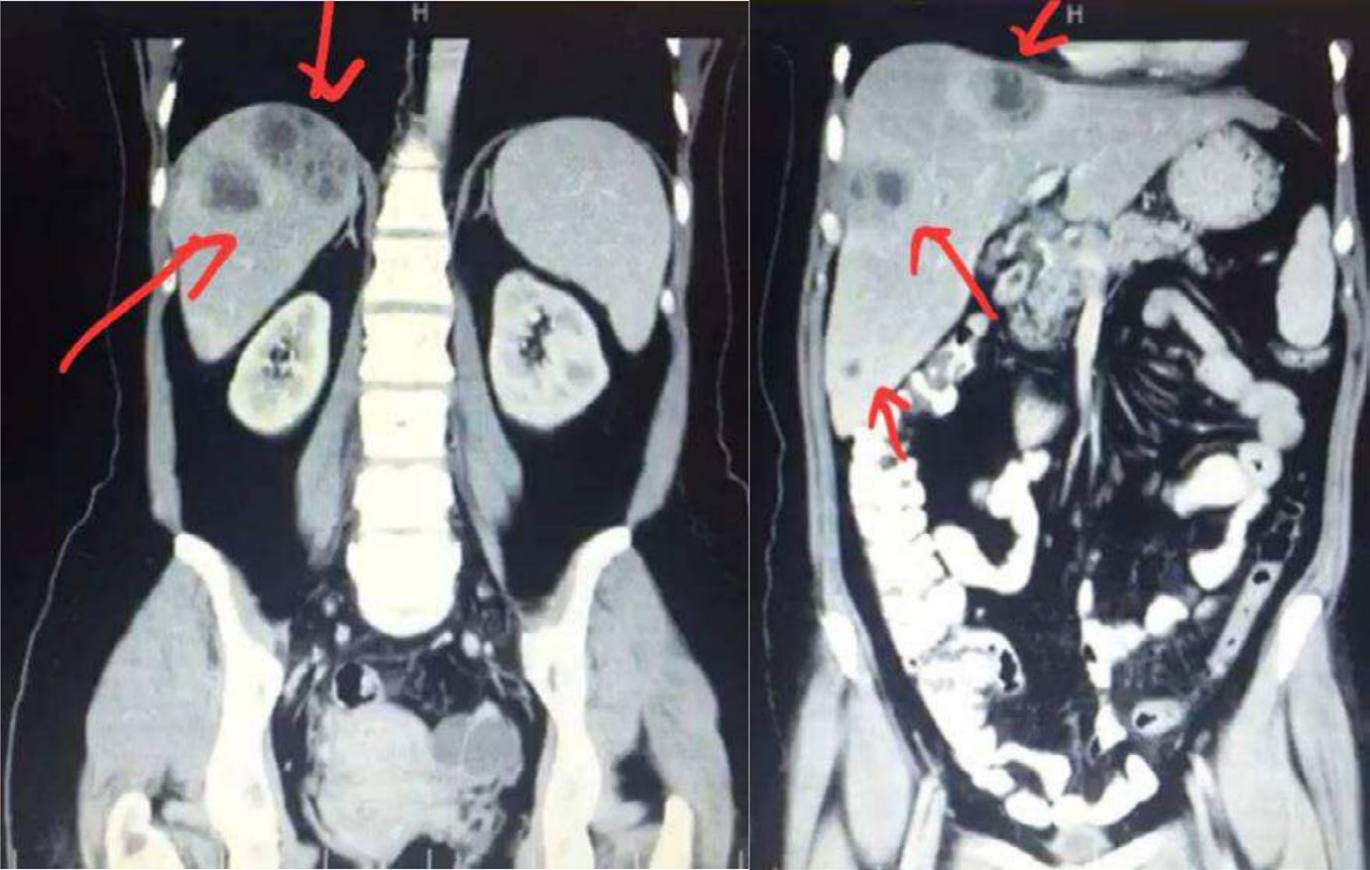
CT scan of abdomen 4 months before admission showing multiple abscesses in the liver with no pleural effusion.

History of present illness: The patient had been experiencing constitutional symptoms of malaise, fatigue, anorexia, and weight loss of about 2 kg, with vague right abdominal pain over the past couple of months. She was found to have multiple liver lesions in ultrasonography and CT scan of the liver 4 months ago, which were suggestive of multiple abscesses versus metastatic liver lesions. The biopsy of the lesions had revealed multiple liver abscesses. Unfortunately, the patient's treatment had been postponed due to the restrictions that the healthcare system was facing during the COVID‐19 pandemic as well as the patient's reluctance to follow up (Figure 1).

Patient chose not to show up until she started experiencing increasing fatigue, restlessness, and myalgia during the week before admission, followed by a sudden onset of severe chest pain with pressure under her right ribs and increasing dyspnea, which started 1 before admission. The pain lasted for 4 h and was associated with nausea and vomiting.

Her past medical history was unremarkable. She denied using any medications on a regular basis. She was married with two healthy children and had no history of smoking, drug use, high‐risk behaviors, recent travel, or contact with sick individuals.

On physical examination, the patient appeared to be in severe respiratory distress. She had tachycardia with a heart rate of 125 beats per minute, a respiratory rate of 26 per minute, oxygen saturation of 88% on room air, and 94% on 6 L per minute of oxygen by nasal cannula. Her blood pressure was 100/70 mmHg, and her temperature was 37.3°C. Her accessory muscles of respiration were recruited. Palpation of the chest did not elicit any tenderness; however, she exhibited right upper quadrant abdominal tenderness. On auscultation, breath sounds were absent in the lower right lung. The patient's weight and height were 63 kg and 165 cm, respectively. The rest of the physical examination was unremarkable.

## 3 Methods

**FIGURE 2:**
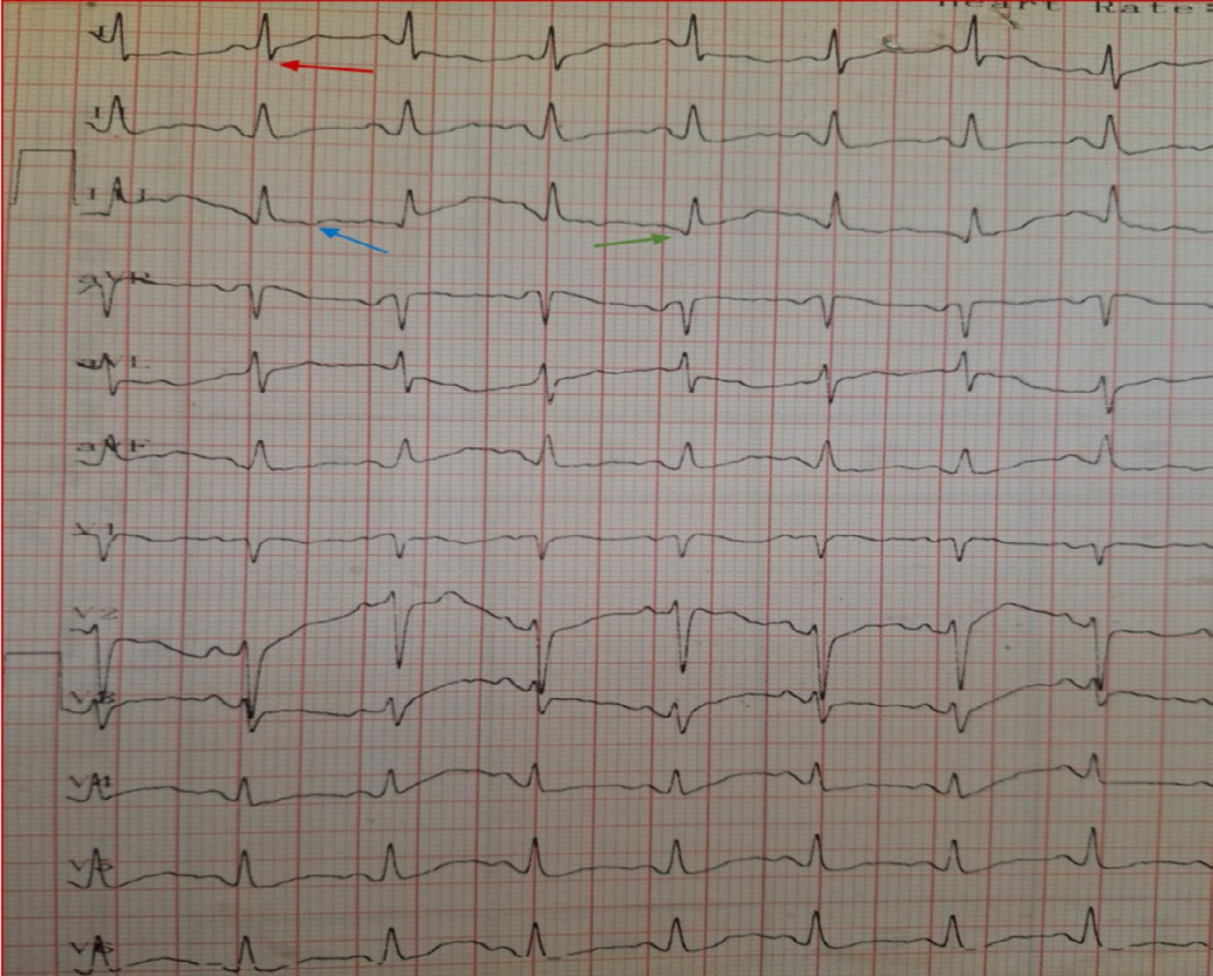
ECG of admission. Showing tachycardia and S1Q3T3 pattern. The abnormal negative S wave on lead I (red arrow), Q wave (green arrow) and negative T wave (blue arrow) on lead III are presented.

The electrocardiograph (ECG) taken in the emergency room showed sinus tachycardia and an S1Q3T3 pattern (Figure 2). A portable chest X‐ray (CXR) revealed mild pleural effusion on the right side. Venous blood gas values were as follows: pH 7.33, pCO2 52 mm (mm) Hg, pO2 17.4 mmHg, HCO3 27.8 mEq/L, and O2 saturation 88% on room air, indicating respiratory acidosis with severe hypoxia. The D‐dimer level was 1199 mg/L (Table 1). The patient had persistent tachycardia and hypoxia with increasing oxygen requirements, leading to her transfer to the intensive care unit (ICU).

**TABLE 1:** Laboratory data. Procalcitonin normal range = 0.5–2 ng/mL. D‐Dimer normal range < 500 mg/L. B/C was conducted 2 days in a row.

Parameter	Value	Parameter	Value
PH	7.33 ↓	Troponin	4.9 ng/L (NL)
PCO_2_	52 mmHg ↑	Procalcitonin	1.8 ng/mL ↑
HCO_3_	27.8 mEq/L ↑	WBC	28,000 /microliter ↑
PO_2_	17.4 mmHg ↓	Neutrophil	85% ↑
HCT	38.4% (NL)	Lymphocyte	9.4% ↓
BE	1.4 mEq/L (NL)	ESR	44 mm/h ↑
Hgb	12.8 g/dL (NL)	PT	12.7 s (NL)
D‐Dimer	1199 mg/L ↑	PTT	37 s (NL)
AST	40 U/L (NL)	BS	135 mg/dL (NL)
ALT	23 U/L (NL)	Cr	0.7 mg/dL (NL)
ALP	537 U/L ↑	Plt	584,000 / mcL units ↑
Bilirubin Total	1.1 mg/dL (NL)	Urea	33 mg/dL (NL)
Bilirubin Direct	0.6 mg/dL (NL)	Na	141 mEq/L (NL)
CPK	30 mcg/L (NL)	K	3.6 mEq/L (NL)
Cholesterol	163 mg/dL (NL)	CRP	I+
HDL	33 mmol/L ↓	U/A	NL
LDL	122 mmol/L ↑	U/C	Candida
TG	135 mg/dL (NL)	B/C × 2	Negative

Abbreviations: ALP, Alkaline phosphatase; ALT, Alanine transaminase; AST, Aspartate transaminase; B/C, Blood culture; BE, Base excess; BS, Blood sugar; CPK, Creatine phosphokinase; Creatine, Cr; CRP, C‐reactive protein; ESR, Erythrocyte sedimentation rate; g/dL, Grams per deciliter; HCT, Hematocrit; HDL, High‐density lipoprotein; Hgb, Hemoglobin; LDL, Low‐density lipoprotein; mcg/L, Micrograms per liter; mcL, Microliter; mEq/L, Milliequivalents per liter; mg/dL, Milligram per deciliter; mg/dL, Milligram per deciliter; mg/L, Milligram per liter; mmHg, Millimeters of mercury; mmol/L, Millimoles per liter; ng/mL, Nanogram per milliliter; NL, Normal; Plt, Platelet; PT, Prothrombin time; PTT, Partial thromboplastin time; TG, Triglyceride; U/A, Urine analysis; U/C, Urine culture; U/L, Units per liter.

**FIGURE 3:**
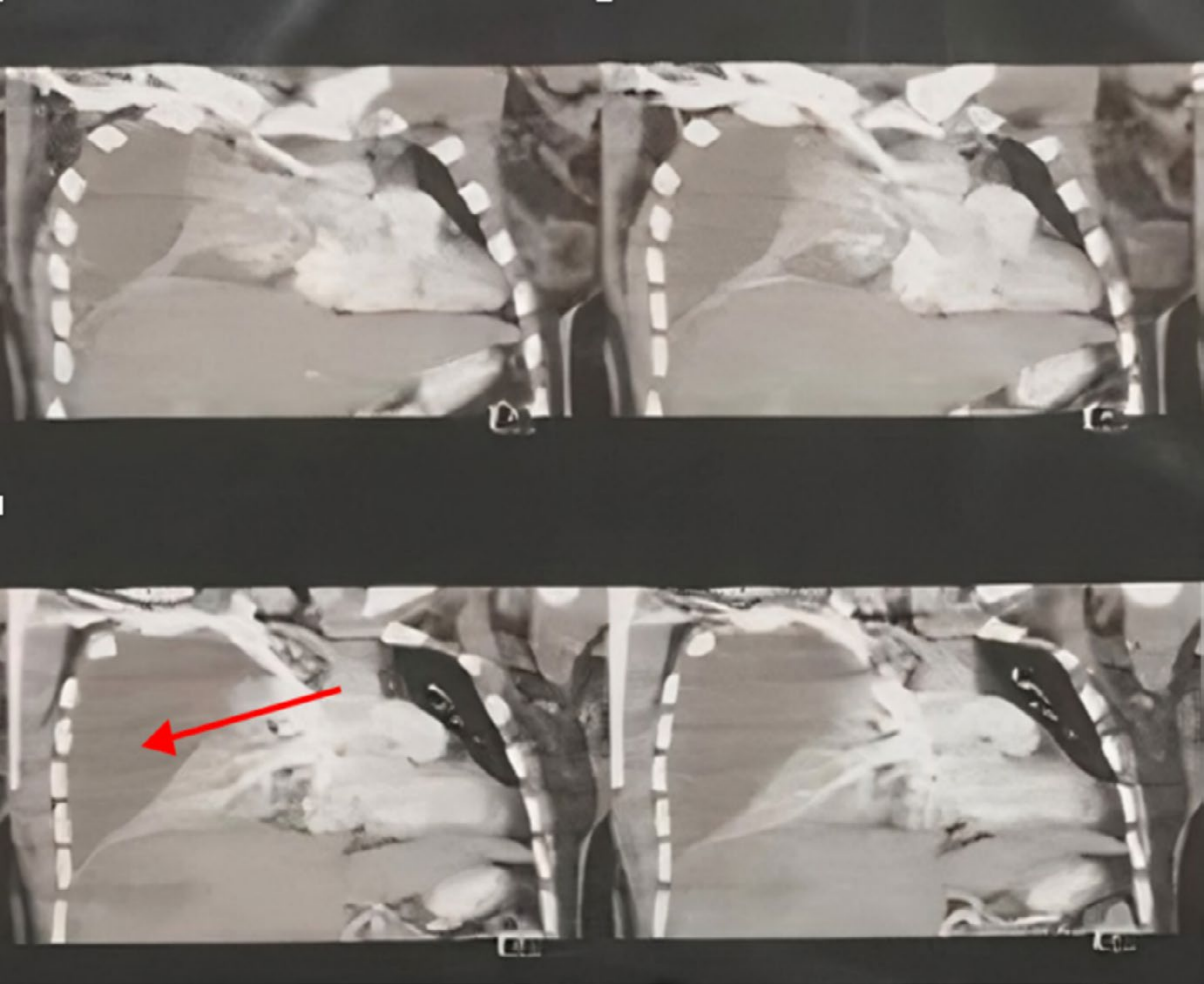
CTPA showing pleural effusion on right hemi‐thorax with shift of mediastinum to the left (red arrow).

The patient's clinical features, along with the ECG findings of tachycardia and S1Q3T3 pattern, with a high D‐Dimer suggested pulmonary embolism as the most likely diagnosis. The patient was treated with therapeutic dose of enoxaparin. She was also given ASA 325 mg and clopidogrel 300 mg on arrival by the ER specialist. A computed tomography pulmonary angiogram (CTPA) was requested; however this was performed 14 h after the patient's presentation to the emergency room. Surprisingly, the CTPA revealed a right‐sided massive pleural effusion and right lung collapse with a shift of the mediastinum to the left, as well as a large mass in the liver and no evidence of pulmonary emboli. The mild pleural effusion had progressed to a massive pleural effusion during 14 h from the presentation. (Figure 3). This was suggestive of the rupture of the liver abscess or the rupture of a simultaneous lung abscess in the context of an untreated liver abscess.

A Doppler ultrasound of the lower extremities was negative for deep venous thrombosis. A 2‐D echocardiogram showed a normal ejection fraction and normal diastolic function. The size of the right ventricle and right atrium was reported to be normal, but the pulmonary artery pressure was not documented in the echocardiography report. PCR for COVID‐19 was negative. Abdominal and pelvic ultrasonography presented a 33 × 47 mm hypoechoic heterogenic mass in the right hepatic segments with irregular borders. It is noteworthy that there was no evidence of pleural effusion on the patient's previous CT scan, taken about 4 months earlier. Blood work showed leukocytosis with a shift to the left. Analysis of the pleural fluid showed exudative fluid with neutrophil predominance (Table 2). As the investigations implied, the patient was diagnosed with an empyema. She was started on broad‐spectrum intravenous antibiotics (meropenem and metronidazole) and an emergent surgical consultation was requested for the insertion of a chest tube, as the patient was in a quite severe respiratory distress.

**TABLE 2:** Serology report of the puss extraction. The values coincide with puss.

Parameter	Value	Parameter	Value
Amylase	39 U/L	WBC	100,000 /microliter
Glucose	10 mg/dL	RBC	2750 /microliter
Protein	6.2 g/dL	Total cell count	102,750 /microliter
LDH	6875 U/L	Neutrophil	96%
Albumin	4.4 g/dL	Lymphocyte	3%
pH	7	Monocyte	1%
Color	Creamy	Culture	Negative after 72 h

Abbreviations: LDH, Lactate dehydrogenase; RBC, Red blood cell; U/L, Units per liter.

**FIGURE 4:**
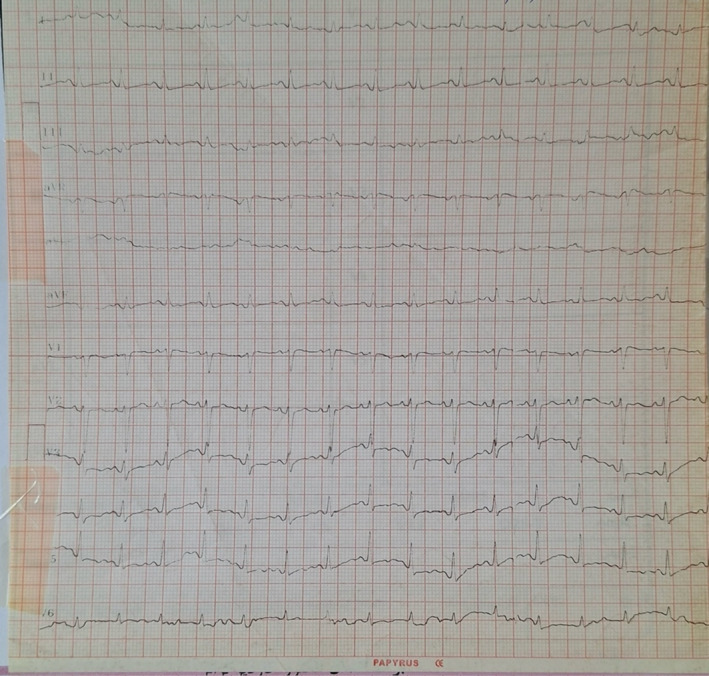
Repeat electrocardiogram showing reversal of S1Q3T3 after drainage of pleural fluid.

The patient underwent chest tube insertion, and 800 cc of pus was removed immediately, followed by drainage of about 2500 mL over the next few days. The patient's dyspnea improved significantly after chest tube insertion. Chest expansion got better, and the S1Q3T3 pattern disappeared on the ECG (Figure 4). The pus drainage was discontinued on the fourth day of admission. Repeat CXR showed that the right costophrenic angle was closed and opacities were present in the middle and lower zones of the right lung, implying pleural adhesion due to empyema.

**FIGURE 5:**
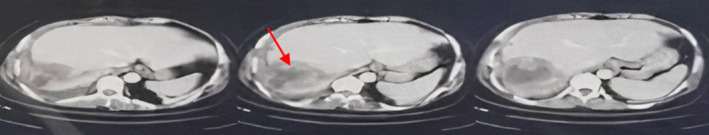
Abdominal CT scan without contrast showing liver abscess (red arrow).

**FIGURE 6:**
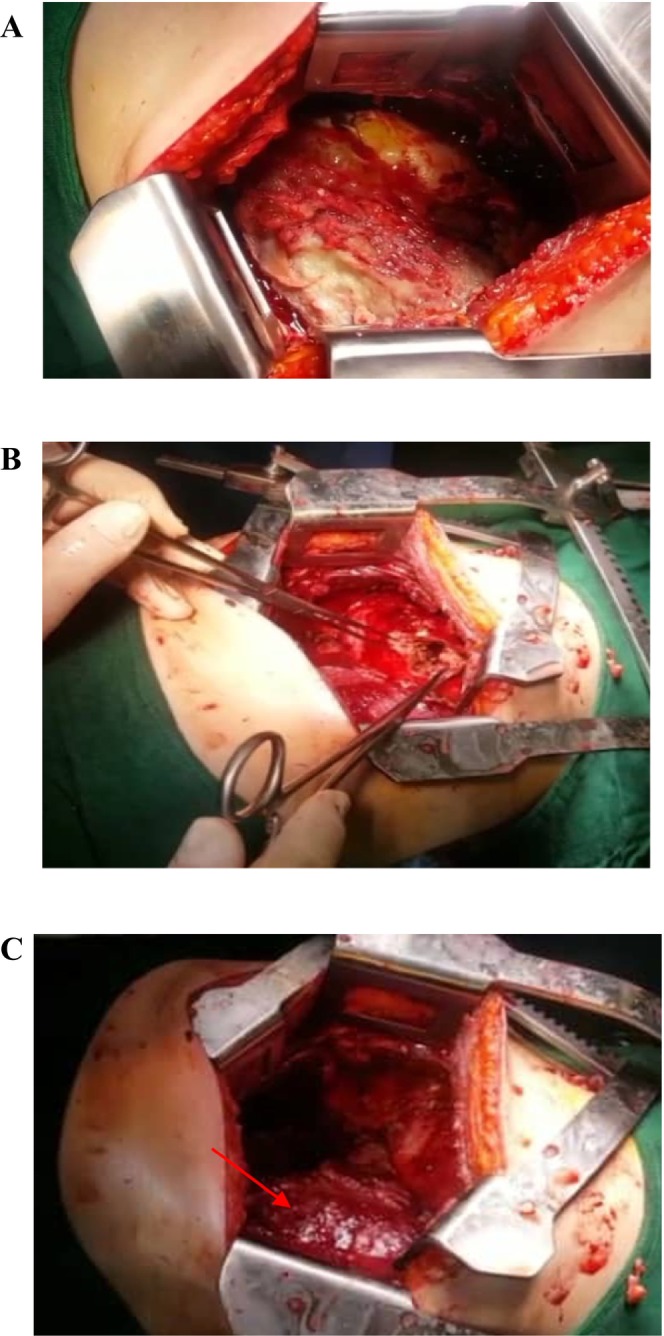
The thoracotomy surgery. (A) The right lung is covered with layers of fibrin and exudates, resulting in lung collapse. (B) Bulging of the right diaphragm at the dome of the liver due to a liver abscess, which was opened up and cleaned by the thoracic surgeon and left open in the pleural cavity to heal. (C) The right lung is seen after the removal of the fibrin exudates (red arrow).

An abdominal CT scan with contrast showed a cystic lesion measuring about 53 × 50 mm in the right liver lobe, favoring abscess formation (Figure 5). Thoracic surgery consultation was requested. Consequently, the patient was transferred to the operating room for the second time on day 7 of her presentation. The surgeon confirmed a pleural abscess, a sub‐diaphragmatic abscess, rib involvement with abscess, and a large liver abscess. The surgeon indicated that the liver abscesses had adhered together, forming a single abscess with one continuous wall. All abscesses were removed from the pleural space, sub‐diaphragmatic space, and liver, along with adhesions, followed by debridement and pleural washing (Figure 6).

## 4 Results

**FIGURE 7:**
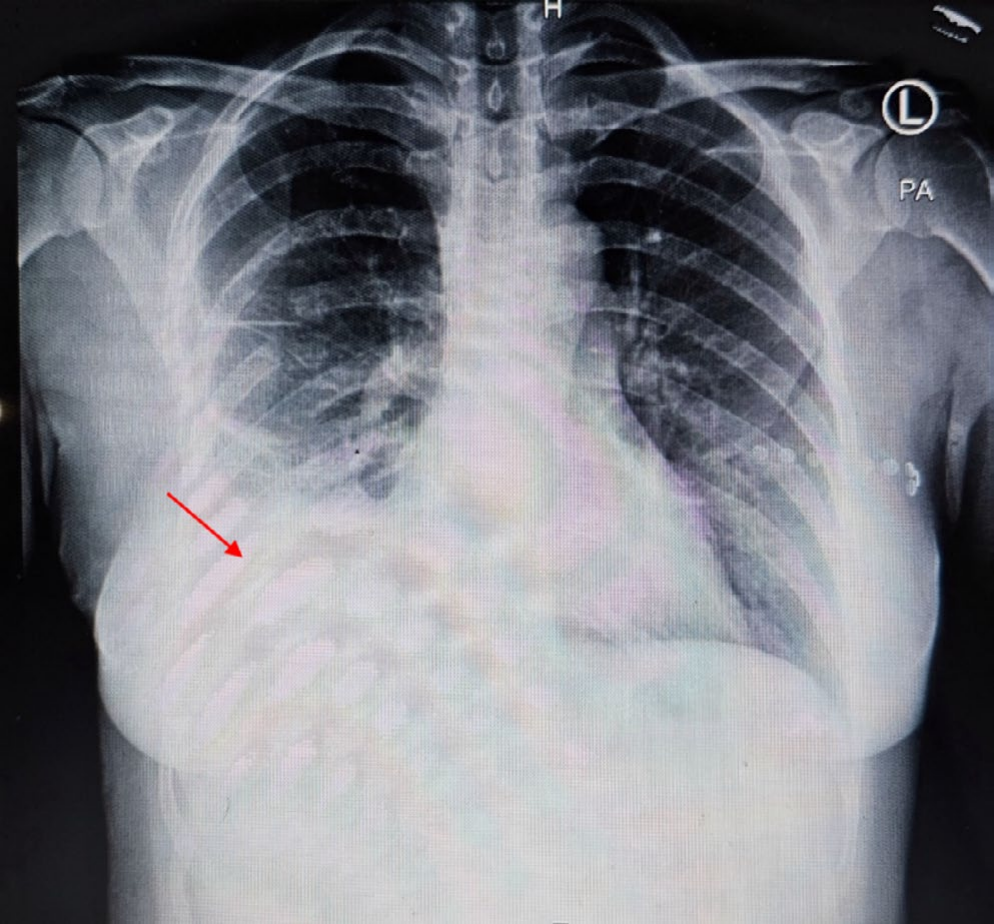
CXR after surgery presenting improvementt in pleural effusion (red arrow).

After the surgery, a collection of serum was visible on ultrasonography, which was extracted through drains. Serology tests for hydatid cyst and *Fasciola hepatica* were negative. According to the pathology report of the surgical sample, a fragment of rib consisted of one piece of bony tissue with attached soft tissue measuring 2 × 1.5 × 1 cm, along with a fragment of fibroconnective tissue and smooth muscle with abscess formation. This suggested that the rib fragment had abscess formation, possibly related to the empyema and pyogenic liver abscess (PLA). A PCR study for *Mycobacterium tuberculosis* was negative. A chest X‐ray 1 week after the surgery showed a reduction in pleural effusion (Figure 7).

Antibiotic therapy continued for 1 month after discharge (discharge antibiotics: Clindamycin and Cefixime). On the discharge day, the symptoms had disappeared. During follow‐ups over 1 year, the patient presented with complete remission of symptoms, with no evidence of pleural effusion or cysts on follow‐up chest X‐rays and abdominal sonography. The primary effusion and abscess were completely cured.

**FIGURE 8:**
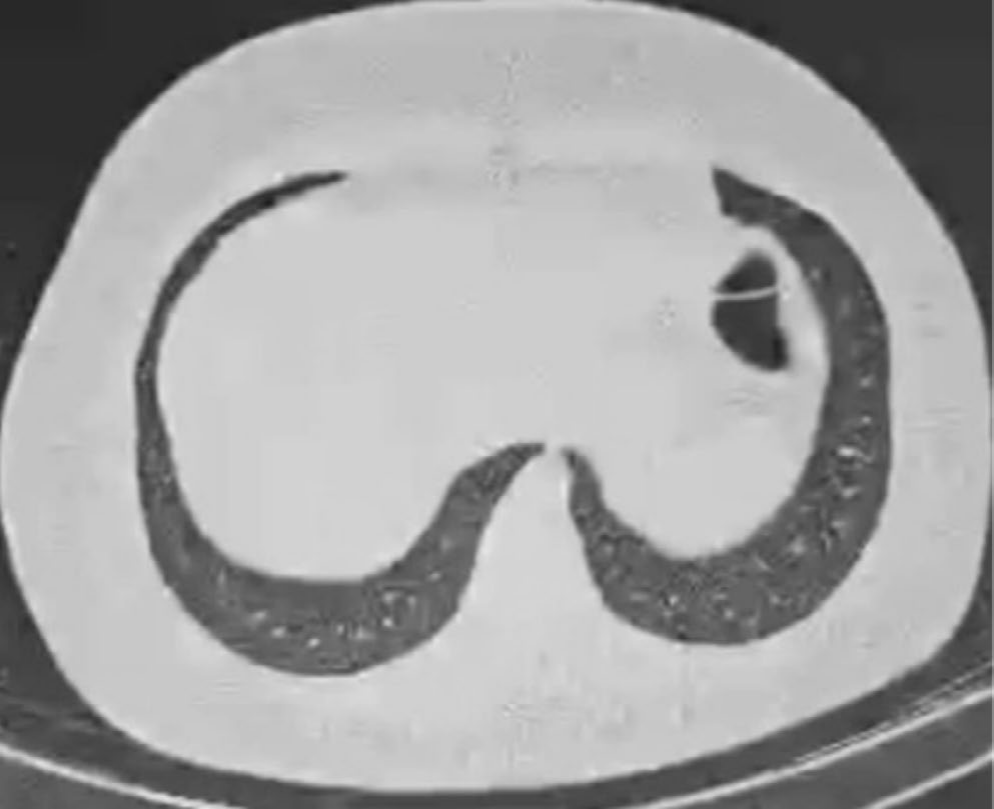
CT scan of the chest after 6 months of treatment showing complete resolution of liver abscesses and pleural effusion.

**FIGURE 9:**
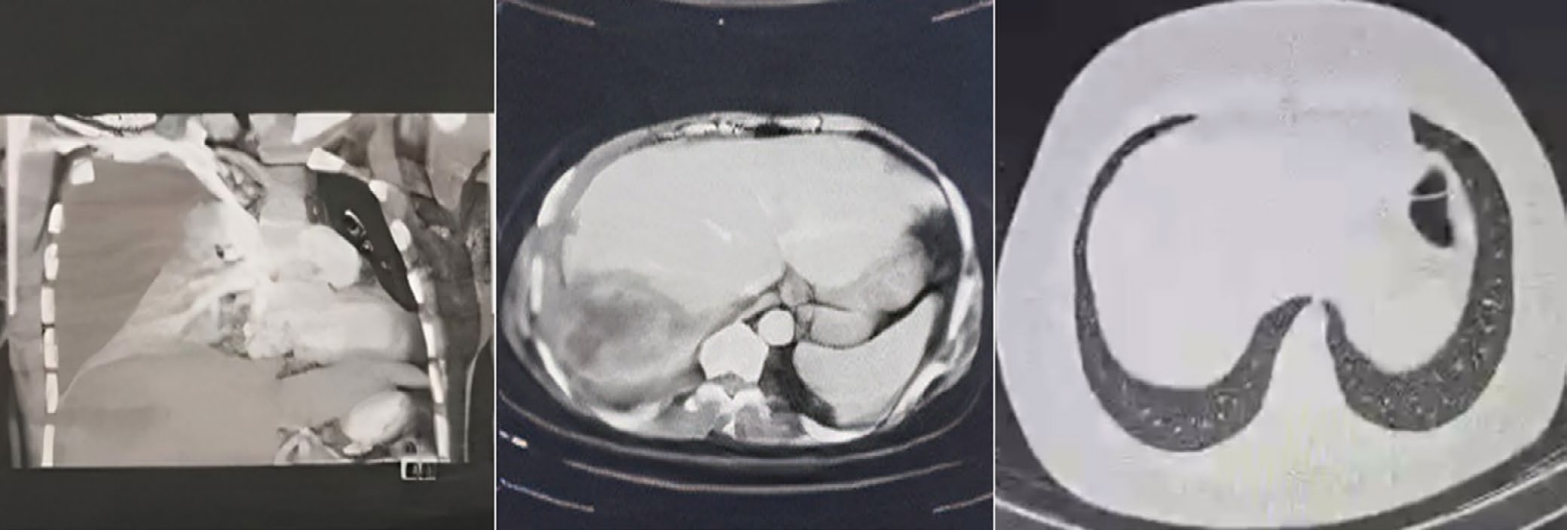
Evolution of chest CT scans from presentation (left), diagnosis (middle), to cure (right).

In summary, the individual was admitted to the hospital after experiencing sudden chest pain, which was later revealed to be caused by the extension of the liver abscess to the chest with the formation of a right lung abscess, causing massive empyema, lung collapse, and mediastinal shift. The primary S1Q3T3 pattern observed on the ECG was attributed to the pressure on the right heart. Chest tube insertion and broad‐spectrum antibiotics failed to remove the pus completely, and she required a partial thoracotomy and decortication to excise the abscess and fibrotic tissues. Over the course of a year‐long follow‐up, the patient experienced complete remission (Figure 8). Detailed information regarding the duration of hospitalization is provided in Table 3 and Figure 9.

**TABLE 3:** summary of the hospital work‐up and results till discharge.

Days findings	Day one (admission to ER)	Day one (admission to ICU)	Day two	Day three (admission to OR)	Day of second surgery and discharge (week one)
CXR	Mild pleural effusion on the right side	/	/	Pleural adhesion due to empyema	Pleural effusion reduction
ECG	Sinus tachycardia and an S1Q3T3 pattern	Sinus tachycardia and an S1Q3T3 pattern	Sinus tachycardia	Sinus tachycardia	Normal
Laboratory results	Respiratory acidosis	Leukocytosis	Pyogenic pleural effusion	Negative blood and puss culture	Normal
Echocardiography	/	Normal	/	/	Normal
Sonography	/	Normal Doppler	A massive right pleural effusion and a severe lung collapse	Cystic lesion on right liver lobe	Normal collection of serum in right liver lobe after surgery
CT	/	/	Pulmonary embolism was ruled out due to CT angiography	Cystic lesion in the abdominal CT scan and air‐fluid level in the right lung favoring of lung cyst	/
Treatment	Possible ACS work‐up	Possible pulmonary embolism work‐up		Chest tube insertion	Removal of the abscess from lung and liver
Results	Refer to CCU	Antibiotic therapy	Refer to OR	Disappearance of S1Q3T3	Full recovery

Abbreviations: ACS, Acute coronary syndrome; CXR, Chest X‐ray; ECG, Electrocardiography; ER, Emergency room; ICU, Intensive care unit; OR, Operating room.

## 5 Discussion

S1Q3T3 on an ECG can help diagnose acute pulmonary embolism, especially if T waves in leads V1 and V2 are inverted in conjunction with the pattern. The S1Q3T3 pattern can be associated with transient right bundle branch block (RBBB) in acute pulmonary embolism and is thought to be caused by acute right ventricular overload and dilatation accompanied by subendocardial ischemia in the right bundle [12]. However, the S1Q3T3 pattern is non‐specific and can occur in the presence or absence of pulmonary embolism even among healthy individuals. In one study from Nigeria, the prevalence of S1Q3T3 among apparently healthy young adults was 0.93% [13]. In a case report of a pregnant asthmatic patient during acute bronchospasm, the S1Q3T3 pattern was observed, suggesting that acute bronchospasm can cause right heart strain and lead to the development of this pattern. The proposed mechanism is dynamic hyperinflation leading to increased right ventricular load and elevated pulmonary artery pressure [5]. On the other hand, the companionship of the PLA with empyema occurs in several possible mechanisms involving the direct spread of infection from the liver abscess to the pleural space through the diaphragm or lymphatic or hematogenous routes. The risk of developing pleural empyema secondary to a liver abscess is significantly increased, with PLA raising the risk of empyema by 18 times [14]. For the companionship of PLA and cardiac complications, only one case report presented a 65‐year‐old male patient admitted with fever, dyspnea, and RUQ abdominal pain. The patient was diagnosed with pleural effusion due to a considerable liver abscess discharge to the pleural cavity. The patient was treated. A few days later, pericarditis and cardiac tamponade developed [15].

In this report, a 41‐year‐old female patient was admitted to the emergency room with a sudden chest pain, dyspnea, cold sweating, vomiting, and an S1Q3T3 pattern on the ECG. The patient's CXR at presentation showed a relatively mild pleural effusion in the right side. At first, the patient was treated with the impression of acute coronary syndrome and/or pulmonary embolism with anti‐platelet and anti‐coagulation medicines. But further investigations showed that the patient's presentations had been due to a rapidly expanding pyogenic pleural effusion as a result of the extension of a large liver abscess. The pleural effusion was shown to have increased massively within about 14 h, as is shown in the CT angiography of the chest, which was initially requested with the impression of pulmonary emboli. The patient was treated with antibiotics and underwent a partial thoracotomy to remove the pus, which led to the disappearance of the S1Q3T3 pattern. Despite initial improvement in symptoms, complications developed, including pleural adhesion, pleural abscess, and lung collapse, which required thoracic surgery. In the case report presented, the patient had a history of multiple liver abscesses 4 months prior, which were left untreated due to COVID‐19 pandemic restrictions and also the patient's non‐compliance. This delay in treatment likely contributed to the development of the confluence of the multiple abscesses to make a huge liver abscess with further extension of the infection to the chest cavity and its complications. Despite the complicated course, the patient was cured and has been completely healthy during the follow‐up visits for 2 years. The result of the chest and abdominal CT scan after 1 year and serial ultrasounds had remained normal. The case presented indicated that the S1Q3T3 pattern can appear under different circumstances other than pulmonary embolism.

Marwah et al. (2020) reported a case involving a 22‐year‐old male with no comorbidities who presented with worsening breathlessness and right‐sided pleuritic chest pain. Initially misdiagnosed with pulmonary embolism due to an S1Q3T3 ECG pattern, further evaluation revealed a massive right‐sided pleural effusion. Treatment included thoracentesis and antibiotics, leading to significant recovery and normalization of the ECG [16]. Similar to our case, both cases emphasize the need for a thorough evaluation and differential diagnosis to avoid misdiagnosis and ensure appropriate treatment. PLAs are a serious condition with potentially fatal consequences [17]. Empyema can be a complication of liver abscesses, whether pyogenic or amoebic, and may necessitate interventions such as chest tube thoracostomy and decortication to prevent recurrence and chronic infection [18]. Furthermore, diaphragmatic defects can serve as a pathway for the spread of intra‐abdominal sepsis to the pleural cavity, potentially leading to empyema [19].

## 6 Conclusion

In conclusion, this case report highlights the importance of considering differential diagnosis for the S1Q3T3 pattern on the ECG. These include all causes of acute cor pulmonale, like pneumothorax, acute bronchospasm, acute lung injury, as well as acute progressive pyogenic pleural effusion, as presented in this case report. Early diagnosis and appropriate management of pleural effusion are essential to prevent complications and to improve patient outcomes.

## Author Contributions

**Mahmonir Mohammadi:** methodology, project administration, resources, supervision. **Mahnaz Valizadeh:** methodology, resources, supervision, writing – review and editing. **Nasrin Rahmani‐ju:** resources, validation. **Yasamin Khosravaninezhad:** conceptualization, methodology, software, writing – original draft, writing – review and editing.

## Ethics Statement

The manuscript ethics were performed following the Declaration of Helsinki; however, ethical approval is not required at Amir‐al‐Momenin Medical School Hospital to publish an anonymous case report.

## Consent

Written informed consent was obtained from the patient to publish this case report and any accompanying images. A copy of the written consent is available for review by the Editor‐in‐Chief of this journal.

## Conflicts of Interest

The authors declare no conflicts of interest.

## Data Availability

All the information used in this manuscript is available in the patient's chart at the center.
